# Supplementary material for: Evidence of spin and charge density waves in Chromium electronic bands
Source: Commun Mater. 2025 Apr 12;6(1):70. doi: 10.1038/s43246-025-00789-0 (PMC11993358; doi:10.1038/s43246-025-00789-0)
Supplement: Supplementary file 2 — Supplementary Information for “Evidence of spin and charge density wave in Chromium electronic bands” [file 43246_2025_789_MOESM2_ESM.pdf]

Supplementary Information for
"Evidence of spin and charge density wave in Chromium electronic bands"

Federico Bisti,[1,2] Paolo Settembri,[1] Jan Minár,[3] Victor A. Rogalev,[2] Roland Widmer,[4] Oliver Gröning,[4] Ming Shi,[2,5] Thorsten Schmitt,[2] Gianni Profeta,[1,6] and Vladimir N. Strocov[2]

[1]*Dipartimento di Scienze Fisiche e Chimiche, Università dell'Aquila, Via Vetoio 10, 67100, L'Aquila, Italy*
[2]*Swiss Light Source, Paul Scherrer Institute, CH-5232 Villigen PSI, Switzerland*
[3]*New Technologies Research Centre, University of West Bohemia, 301 00 Pilsen, Czech Republic*
[4]*nanotech@surfaces Laboratory, EMPA, Swiss Federal Laboratories for Materials Science and Technology, Ueberlandstrasse 129, 8600 Duebendorf, Switzerland*
[5]*Center for Correlated Matter and School of Physics, Zhejiang University, 310058 Hangzhou, China*
[6]*CNR-SPIN L'Aquila, Via Vetoio 10, 67100 L'Aquila, Italy*

Supplementary Note 1. DFT Calculations

Our density functional theory calculations were performed using different xc-functionals, such as the PBE [1] and PBESOL [2] GGAs, and the SCAN [3] meta-GGA. While the modifications on the Fermi surface in the non-magnetic phase have been already investigated in great detail for different xc-functionals [4], we were mainly interested in the AF and SDW phases, obtaining very different predictions for the magnetic moment of the Cr atoms [4–7]. While the LDA computations underestimate the magnetic moment with respect to the experimental value, the GGAs and especially the meta-GGAs calculations overestimate it [4]. All these failures, and the SDWs not being predicted to be the ground state by any of these DFT calculations, can be connected to the fact that local and semi-local approximations for the xc-functionals do not account explicitly for non-local AF correlations. In the work of Capelle *et al.* [8] methods for a correct description of AF and SDW systems are investigated, suggesting LDA as the starting point to construct related proper functionals. In our work, we decided to emulate the effect of such functionals adopting the DFT+U in the Dudarev *et al.* [9] formulation. The Hubbard term U is then tuned to match the experimental magnetic moment of the Cr atoms. A similar approach was proposed for pnictides [10] where, in order to reduce the predicted magnetic moment value by the PBE and SCAN calculations, a negative U value was applied. In our case, by using the LDA+U formulation on the AF configuration, we went from the starting magnetization value of 0.31 μ_B (U=0, so for LDA), to the experimentally measured value of 0.6 μ_B by using U=0.08 eV, see Supplementary Figure 1. Analogously, for the SDW configuration, the tuned U value is U=0.13 eV, obtaining the correct sinusoidal magnetic moment profile, with an amplitude of 0.6 μ_B, as shown in Supplementary Figure 6. Like in previous works [5–7], the SDWs created using such procedures are still not the ground state of the DFT calculations, but are nonetheless found as stable solutions when starting with the initial magnetization guess like previously described.

Supplementary Note 2. Unfolding Procedure

The concept of a supercell is very useful in *ab-initio* simulations, giving the possibility of studying different configurations, like surfaces, magnetic orderings, charge and spin density waves. This large cell is needed for reproducing long range effects that are usually small perturbation on the original system. Therefore, to compare this information with the experiments, it is often needed a procedure to unfold the electronic states from the (smaller) supercell BZ into the (larger) original one [11–17].
In our case the non-magnetic bulk Cr system has a body-centered-cubic (BCC) unit cell. Instead, to describe the AF configuration, a simple cubic (SC) cell is required, where the basis is composted by two Cr atoms with equal and opposite magnetization. Since the SC cell has double the volume of the BCC one, its BZ is half the volume of the initial one; they are shown in the (c) panel of Fig. 1 of the main manuscript, in light grey (BCC) and pale pink (SC). The unfolding procedure consists in projecting the Kohn-Sham wavefunctions for the AF-phase SC system onto a set of states chosen as basis, in our case the 3d-orbitals of Cr, in the BCC BZ, allowing us to reconstruct the band structure and FS of the system, with the value of the projections giving us an indication of the spectral weight of such states, which is comparable to ARPES experiments. In the same way the states obtained from the DFT simulation of the SDW supercell, shown in Fig. 1 (a) of the main manuscript, are projected onto the BCC BZ; giving us a possible comparison with the ARPES data obtained on the Cr sample.
To better understand the unfolding behaviour and the weights that we obtain from it, we studied the unfolding of an AF-phase SC cell of Cr as a function of the magnetization value, which we tune using the Hubbard parameter U. The results of the band structure unfolding for different U values are shown in the (a,b,c,d,e,f) panels of Supplementary Figure 1. We can see the effects of the different magnetization values on both the intensities and band gaps of the band structure. We try to quantify this behavior through the unfolding procedure and our results are shown in the (g) panel of Supplementary Figure 1, where the ratio between the replicas and pri-

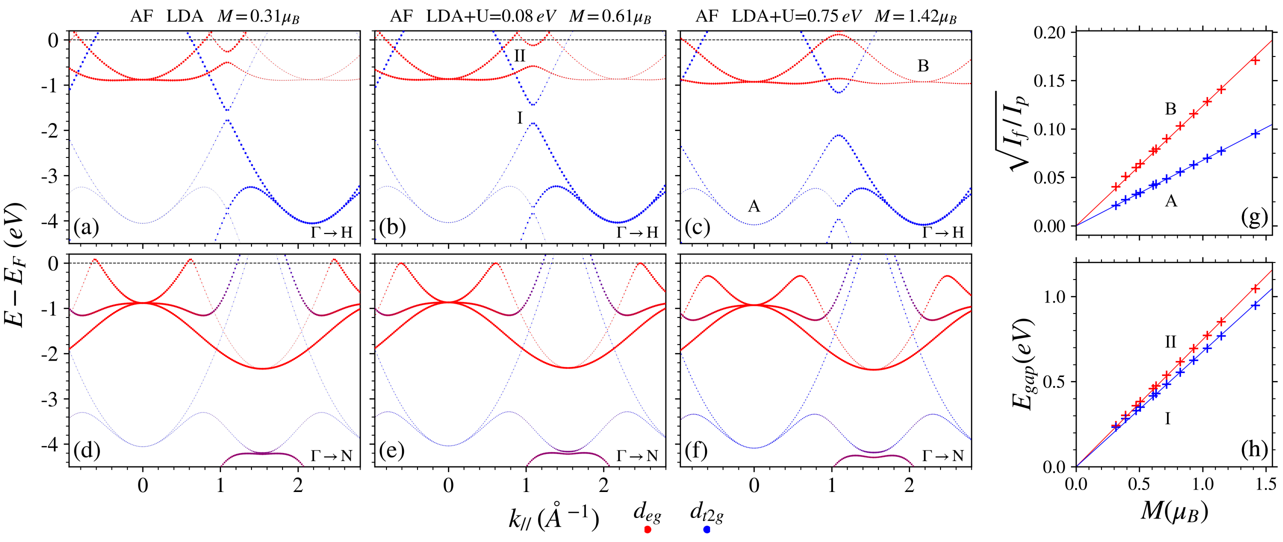

Supplementary Figure 1. **Unfolded bands at different magnetizations.** Unfolding of the band structure (Γ-H in the (a,b,c) panels, Γ-N in the (d,e,f) panels) of the Cr AF phase, obtained in LDA+U for different U values: (a,d) U=0 eV, M=0.31 $\mu_{\rm B}$, (b,e) U=0.08 eV, M=0.61 $\mu_{\rm B}$, (c,f) U=0.75 eV, M=1.42 $\mu_{\rm B}$. The colors represent the projection of the states onto the e_g (red) and t_{2g} (blue) representations, while the dot intensity and size are related to the weight of the state, given by the unfolding procedure. (g) Intensity ratio between the AF ordering replicas, indicated with A and B in panel (c), and the primary signal at Γ and H as a function of the magnetization. (h) Band gaps energy at the Bragg plane present along the Γ-H direction, indicated with I and II in panel (b), as a function of the magnetization.

mary signals at Γ and H, is reported as a function of the magnetization, showing a quadratic growth whose slope depends on the related band. We expect that for large magnetizations the ratio saturates to 1 as reasonable. We can see in the (h) panel of Supplementary Figure 1, how the band gaps, present half-way along the Γ-H direction, change as a function of the atoms magnetization M. In agreement with the Hartree-Fock expectation of the band gap opening, it grows linearly with the magnetization.
We can understand the behaviour of the unfolding weight as a measure of how non-equivalent the two Cr atoms are in the simple cubic cell: for zero magnetization they are equivalent and the weight of the replicas is null; by increasing the magnetization (through the U), they become more and more diverse and the weight increases.
We now try to derive a fit function for the unfolding weight in the AF system, starting from the work of S. Kurth and F.G. Eich [18], in which they studied a uniform electron gas with a SDW in the DFT formalism. From the solutions of the Kohn-Sham equation:

$$\left(-\frac{\nabla^2}{2}+\mu_{\rm B}\sigma\mathbf{B}(\mathbf{r})\right)\Phi_i(\mathbf{r})=\epsilon_i\Phi_i(\mathbf{r}) \qquad \text{(SE1)}$$

with $\mathbf{B}(\mathbf{r})=[\mathrm{B}\cos{(qz)},\mathrm{B}\sin{(qz)},0]$, which are reported in the paper [18], we evaluated how the unfolding coefficient would behave for such a system, obtaining:

$$n(\kappa)=\frac{1}{2}\left[1\pm\frac{1}{\sqrt{1+4\frac{c^2}{\kappa^2}}}\right], \qquad \text{(SE2)}$$

where $\kappa=k-\frac{q}{2}$, $c=\frac{\mu_{\rm B}\mathrm{B}}{q}$.
We used this result as a starting point to describe theoretically the intensity of the unfolding of our Cr system, fitting the data with the function:

$$f(k;c,a)=\frac{a}{2}\left[1\pm\frac{1}{\sqrt{1+4\frac{c^2}{\kappa^2}}}\right]. \qquad \text{(SE3)}$$

The a parameter, which indicates the maximal value of the coefficients, takes into account the form factor of the specific band we are considering, which are not present in the free electron example; while the c parameter indicates the spread of the unfolding weights. It can be seen in Supplementary Figure 2 how the spread increases for higher magnetization values, and in particular it increases linearly with the magnetization (which is in agreement with the model of Ref. [18] for which c is proportional to B, and so to M). A small variation of the c fitting parameter is also present between different bands, showing the limits of this approach. Small quantitative deviations should also be expected since the unfolding is performed by projecting onto a non-complete set of atomic orbitals.

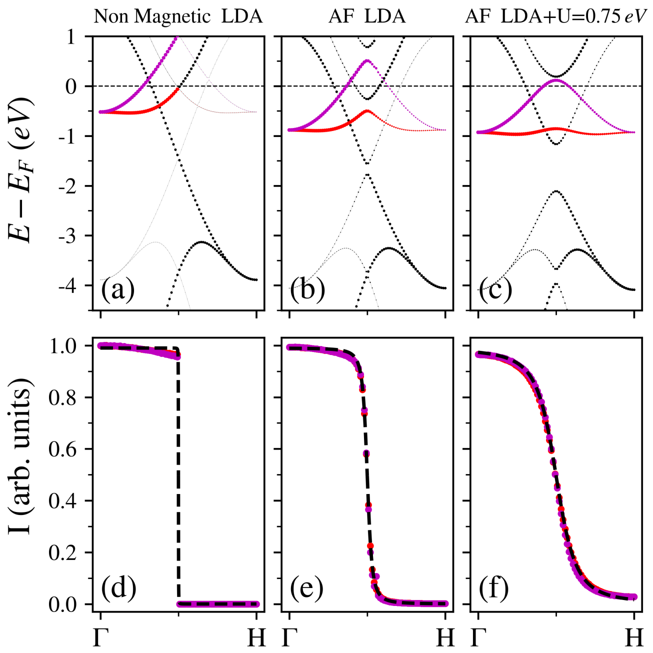

Supplementary Figure 2. **Unfolding weight behavior.** The electronic band structures along the Γ-H direction are shown for a non-magnetic LDA (a), for an AF phase LDA (b) and an LDA+U=0.75 eV computation (c). The color of the bands is a result of the unfolding procedure on the BCC primitive cell, with two specific bands being highlighted in red and magenta and taken as a reference. The (d,e,f) panels show the intensity of the projection of the states of such two bands along the Γ-H direction (in red and magenta). The dashed black lines show a fit of such intensities obtained using $f(k; c, a)$.

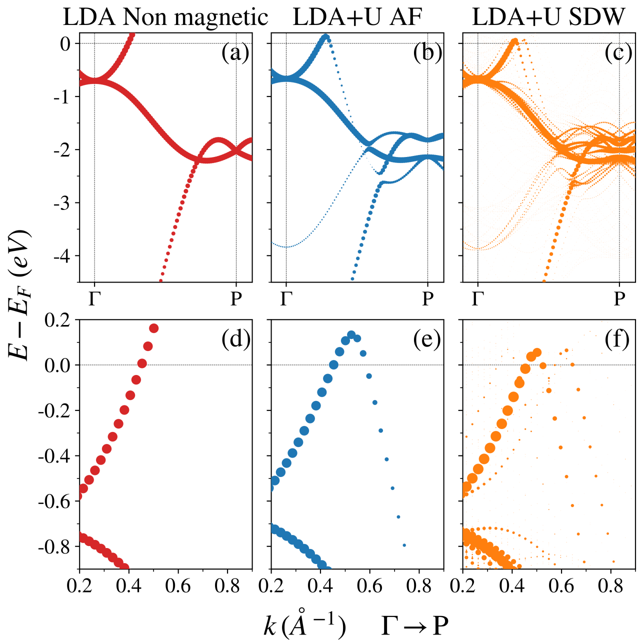

Supplementary Figure 3. **Unfolded Γ-P band structure.** Panels (a,b,c) show the unfolded band structures along the Γ-P direction for different magnetic configurations, with a zoom near the Fermi level reported in panels (d,e,f). The band structures are shown for a LDA non-magnetic BCC unit cell (a,d), for a LDA+U AF SC cell (b,e) and for a LDA+U 21 unit cell SDW (c,f). The AF calculation adopts U=0.08 eV, with an atomic magnetic moment of M≃0.6 μ_B, while the SDW one uses U=0.13 eV, with a M≃0.6 μ_B maximum magnetization. A rigid shift of +0.2 eV was adopted.

Supplementary Note 3. Additional Band Structures

In this section, we expand the exploration of the Chromium band structure by offering additional theoretical and experimental data on other high-symmetry directions.
The first one is the Γ-P direction, which is very peculiar since independently from the SDW orientation, all the P points are equivalent. The Γ-P directions correspond to the {111} directions while the SDWs propagate along the {100} directions, thus the angle between them is always 45°. This brings to the conclusion that, regardless of the crystal or SDW orientation, the band structure along the Γ-P direction is always the same.

Due to the geometry of our experimental setup, such direction could not be probed directly, but we can compare our theoretical calculations with the experimental measures already present in the literature [19]. In Supplementary Figure 3 we report the unfolded band structures for different magnetic configurations, with a zoom of the bands near the Fermi level. An excellent agreement between our theoretical results and the ARPES measurements shown in Fig. 9 of Ref. [19] can be recognized. In particular, the panel Fig. 9 (a) of Rotenberg *et. al*, reporting the data of a commensurate SDW (C-SDW), is equivalent to our panel (e), which corresponds to the AF configuration in our work. Their panel Fig. 9 (b), reporting the incommensurate SDW (IC-SDW), is equivalent to our panel (f) perfectly reproducing the double backfolding of the band, which is the most relevant feature of the SDW configuration.
Another high-symmetry direction of the system is the N-H direction, for which we report the experimental ARPES data and the unfolded band structure in Supplementary Figure 4. The ARPES data shown in panels (a) and (f) come from two different measurements. In panel (a) the measure performed using 638 eV is shown, this corresponds to a Γ plane perpendicular to the surface normal direction, knowing that for Cr (100) the SDW propagates perpendicularly with respect to the surface, the bands probed in panel (a) belong to the plane perpendicular to the SDW (green triangle of Fig. 1 (c) of the main manuscript). The data in panel (f) has been obtained by interpolating the ARPES measurements obtained with a varying photon energy, thus moving in the k_z direction. For the same reason, this will correspond to the plane parallel to the SDW direction (yellow triangle in Fig. 1 (c) of the main manuscript). While the repli-

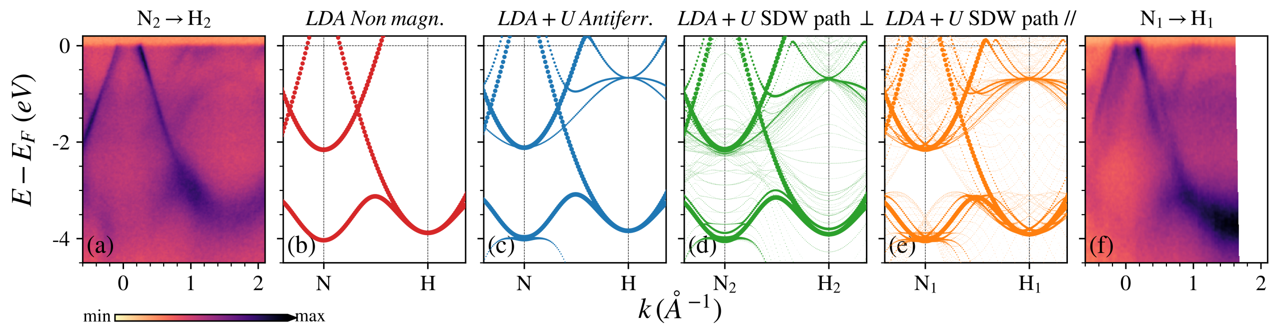

Supplementary Figure 4. **N-H ARPES and Unfolded bands.** Panel (a) shows the band structure along the N-H direction probed using 638 eV photons with circular polarization; this direction lies on the Γ plane perpendicular to the surface normal direction. Panel (f) shows the band structure along the N-H direction obtained from the dataset reported in Fig. 5 (a) of the main manuscript, with N at $k_z \simeq 16.4$ Å^{-1} and H at $k_z \simeq 15.2$ Å^{-1}, using circular polarization. This direction has a 45° tilt with respect to the surface normal direction. Panels (b,c,d,e) show the DFT unfolded band structures for different configurations: (b) LDA non-magnetic BCC unit cell, (c) LDA+U AF SC cell with U=0.08 eV, (d-e) LDA+U 21 unit cell SDW, with U=0.13 eV, along a path that is orthogonal (parallel) to the SDW propagation direction respectively.The experimental data in panels (a,f) are reported using a square root color scale. A rigid shift of +0.2 eV on the DFT bands was adopted.

cas originating from the antiferromagnetic ordering can be clearly seen in both ARPES maps (for example the bands at H near the Fermi energy), features induced by the SDW cannot be easily pointed out. This is also due to the measures being performed with circular polarization instead of linear s and p polarizations, not allowing for a separation of the symmetrical and asymmetrical components. However, we can extrapolate very important information from this comparison: if we look at the linear bands near the N point of panel (f) we can see additional features, that are not present in panel (a). Such features correspond to replicas of the linear bands near the N point rigidly shifted along the k_z component. In fact, one replica lies closer to N, and the other one is further away from the main signal. This behavior does not match any of the possible magnetic configurations and is instead consistent with final state effects, as already pointed out in Fig. 5 of the main manuscript and further discussed also in the following section.

Supplementary Note 4. More Insights on Final State Effects

In this section we present additional experimental data to support the assignment of the replica around H (seen in Fig. 5 of the main manuscript) to multiple scattering effects on the photoemitted electron final-state rather than the magnetic order.

In Supplementary Figure 5 (a,b,c) we report iso-energy ARPES maps at 0 eV, -0.2 eV and -0.6 eV, along the Γ-H direction perpendicular to the crystal surface from the same dataset of Fig. 5 of the main manuscript (up to 1100 eV with circular polarization) with the addition of the 1100-1300 eV photon range using linear $p-$polarization (since such photon energy range was not available with circular polarization). Panels (d,e,f) show the same maps but along the plane parallel to the crystal surface, using 638 eV photons and circular polarization. Along the different iso-energy maps, the square contour replica around H_{870} (H point found using 870 eV photons, indicated in panel (c)), has an intensity similar to the original signal at a distance from it that stays constant in k_z when moving in energy. This results in a square rigidly shifted in k_z which is precisely expanding as the main signal by increasing the binding energy. As already discussed in the main text and reported again in Supplementary Figure 5 (d,e,f), the magnetic order folded signal expands when the main signal contracts (the square around Γ) or vice-versa (the square around H). For this reason, the origin of the square contour replica around H_{870} is from multiple scattering process rather than from the magnetic order. To emphasize this point even more, in panels (g) and (h) of Supplementary Figure 5, we report the band structure along the in-plane Γ_{638}-H_{638} direction (panel (g)) and the out-of-plane Γ_{638}-H_{870}-Γ_{1130} direction (panel (h)). By the comparison between the two panels, we can recognize analogues rigidly k_z shifted replicas coming from multiple scattering process, as already observed along the N-H directions (Supplementary Figure 4): in particular at around 16.2 Å^{-1} and -0.25 eV or -4 eV. It is worth noting how they are not so visible at 14.2 Å^{-1}, even if this region corresponds to a lower photon energy, highlighting the non-trivial behavior of final state effects in photon energy.

The bands presented in panels (g) and (h) should be compared with the DFT data reported in Fig. 3 (b,h) and (c,i) of the main manuscript respectively. The SDW should manifest as double band gaps, smaller in energy with respect to the probed one along the in-plane direction. We must acknowledge, though, that the negative impact of the multiple scattering process on the k_z resolution and the overall signal resolution derived from the interpolated data compromises such identification.

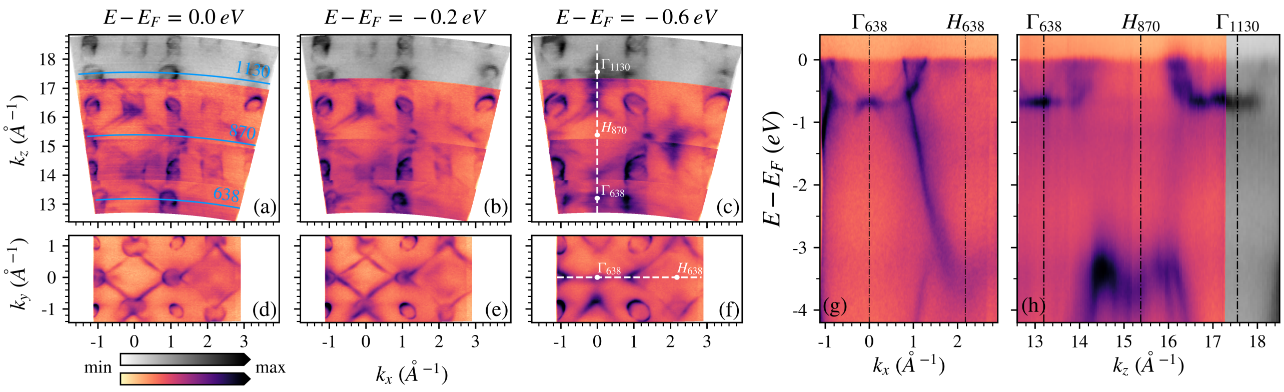

Supplementary Figure 5. **In-plane and out-of-plane ARPES measurements.** (a,b,c) Experimental iso-energy ARPES maps at 0 eV, -0.2 eV and -0.6 eV, along the Γ-H direction perpendicular to the crystal surface, obtained using a photon energy range of 590-1300 eV (590-1100 eV with circular polarization and 1100-1300 eV with linear p-polarization). Panels (d,e,f) show the iso-energy ARPES maps at 0 eV, -0.2 eV and -0.6 eV, along the plane parallel to the crystal surface using 638 eV photons and circular polarization. In panels (c) and (f) the path and the high-symmetry points considered for the band structures reported in panels (h) and (g) respectively, are highlighted in white. Panel (g) shows the band structure for the Γ-H direction moving in the in-plane component k_x while panel (h) shows them moving in the out-of-plane $k_z$component. All the experimental data are reported using a square-root color scale. The data obtained using a linear p-polarization is presented using a gray scale colormap.

Supplementary Note 5. Charge Density Wave Unfolding

We can characterize the SDW+CDW configuration, obtained by relaxing the atoms after the SDW was induced in the system, using several quantities: the atoms displacement with respect to their equilibrium positions; the lattice spacing, which is computed as the average distance between a Cr atom and the two nearest neighbours; the atoms magnetic moments and finally the charge associated to the single atoms. All these quantities are reported in Supplementary Figure 6. We can see how the charge, the lattice spacing and the displacement all show a sinusoidal behaviour with a period half the magnetic moment one.

The lattice spacing, due to the strain wave, is greater in correspondence of atoms with higher magnetic moment, matching the picture predicted for Cr in Ref. [20].

The displacement amplitude, which is ~ 0.008 Å is also perfectly in line with the one estimated by several works [20–22].

If we then compare the charge modulation, which has an amplitude of 0.0045 e^-, with the magnetic moments, we see how the higher charge density corresponds to the SDW nodes; this implies a π phase-shift between the SDW and CDW, which has been discussed in the main manuscript.

Considering a possible incomplete description of the CDW phase, we artificially enhanced the distortion by a factor of $\simeq 10$ to point out the effect of the sole CDW on the band structure. In Supplementary Figure 7 we report the unfolded, non-magnetic, band structure of such system, compared with the ARPES spectra. While band gaps and magnetic replica are clearly absent, several new features appear compared with the non-magnetic bands of Fig. 2 (b,e,h,m) of the main manuscript, which are clearly observed also in our ARPES data, in particular, several new replica bands around the Γ-point in panel (h) of Supplementary Figure 7.

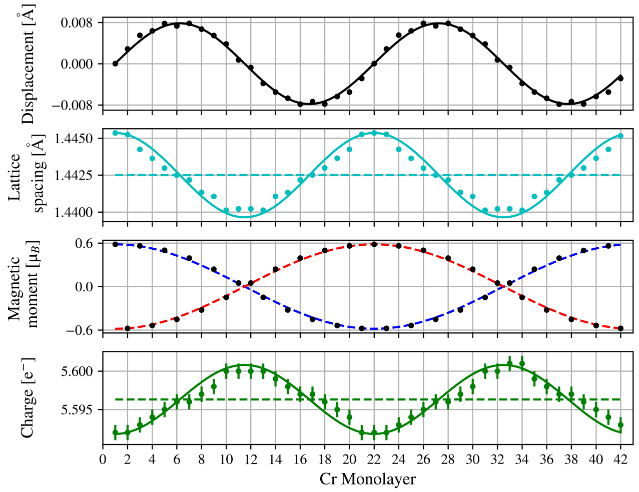

Supplementary Figure 6. **SDW and CDW coexistence.** Displacement, lattice spacing, magnetic moment and charge, associated to the Cr atom in the SDW+CDW system.

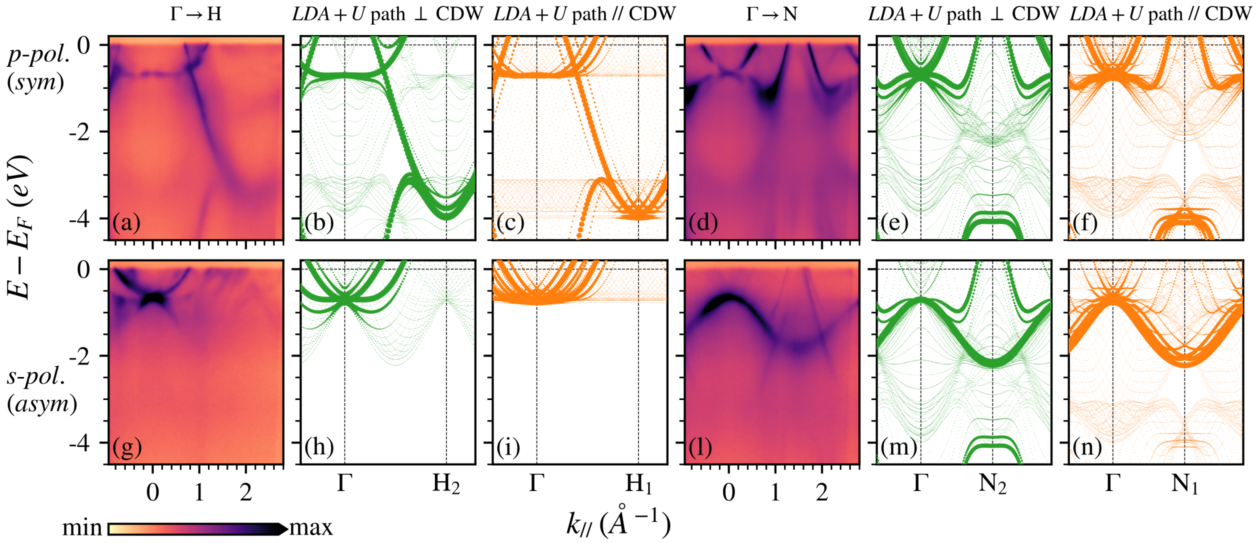

Supplementary Figure 7. **CDW features in the band structure.** (a,d) and (g,l) panels show the band structures along the different directions in the reciprocal space probed using 638 eV photons with $p(s)$-polarization respectively. Panels (b,c,e,f,h,i,m,n) show the unfolded LDA+U band structures for a 21 unit cell non-magnetic CDW system. (b,e,h,m) and (c,f,i,n) panels show the band structures obtained along a path that is orthogonal (parallel) to the CDW propagation direction: green (yellow) triangle in Fig. 1 (c) in the main manuscript). The bands have been decomposed according to their symmetry. A rigid shift of +0.2 eV was adopted.

Supplementary References

[1] John P. Perdew, Kieron Burke, and Matthias Ernzerhof. Generalized gradient approximation made simple. *Phys. Rev. Lett.*, 77:3865–3868, Oct 1996.

[2] John P. Perdew, Adrienn Ruzsinszky, Gábor I. Csonka, Oleg A. Vydrov, Gustavo E. Scuseria, Lucian A. Constantin, Xiaolan Zhou, and Kieron Burke. Restoring the density-gradient expansion for exchange in solids and surfaces. *Phys. Rev. Lett.*, 100:136406, Apr 2008.

[3] Jianwei Sun, Adrienn Ruzsinszky, and John P. Perdew. Strongly constrained and appropriately normed semilocal density functional. *Phys. Rev. Lett.*, 115:036402, Jul 2015.

[4] E. I. Harris-Lee, A. D. N. James, and S. B. Dugdale. Sensitivity of the fermi surface to the treatment of exchange and correlation. *Phys. Rev. B*, 103:235144, Jun 2021.

[5] R. Hafner, D. Spišák, R. Lorenz, and J. Hafner. Magnetic ground state of cr in density-functional theory. *Phys. Rev. B*, 65:184432, May 2002.

[6] R Hafner, D Spisák, R Lorenz, and J Hafner. Does density-functional theory predict a spin-density-wave ground state for cr? *Journal of Physics: Condensed Matter*, 13(11):L239, mar 2001.

[7] S Cottenier, B De Vries, J Meersschaut, and M Rots. What density-functional theory can tell us about the spin-density wave in cr. *Journal of Physics: Condensed Matter*, 14(12):3275, mar 2002.

[8] K. Capelle and L. N. Oliveira. Density-functional theory for spin-density waves and antiferromagnetic systems. *Phys. Rev. B*, 61:15228–15240, Jun 2000.

[9] S. L. Dudarev, G. A. Botton, S. Y. Savrasov, C. J. Humphreys, and A. P. Sutton. Electron-energy-loss spectra and the structural stability of nickel oxide: An lsda+u study. *Phys. Rev. B*, 57:1505–1509, Jan 1998.

[10] Johannes Ferber, Yu-Zhong Zhang, Harald O. Jeschke, and Roser Valentí. Analysis of spin-density wave conductivity spectra of iron pnictides in the framework of density functional theory. *Phys. Rev. B*, 82:165102, Oct 2010.

[11] Wei Ku, Tom Berlijn, and Chi-Cheng Lee. Unfolding first-principles band structures. *Physical review letters*, 104:216401, 05 2010.

[12] F. Bisti, V. A. Rogalev, M. Karolak, S. Paul, A. Gupta, T. Schmitt, G. Güntherodt, V. Eyert, G. Sangiovanni, G. Profeta, and V. N. Strocov. Weakly-correlated nature of ferromagnetism in nonsymmorphic cro_2 revealed by bulk-sensitive soft-x-ray arpes. *Phys. Rev. X*, 7:041067, Dec 2017.

[13] Matteo Jugovac, Cesare Tresca, Iulia Cojocariu, Giovanni Di Santo, Wenjuan Zhao, Luca Petaccia, Paolo Moras, Gianni Profeta, and Federico Bisti. Clarifying the apparent flattening of the graphene band near the van hove singularity. *Phys. Rev. B*, 105:L241107, Jun 2022.

[14] Sara G Mayo, Felix Yndurain, and Jose M Soler. Band unfolding made simple. *Journal of Physics: Condensed Matter*, 32(20):205902, feb 2020.

[15] Paulo V. C. Medeiros, Sven Stafström, and Jonas Björk. Effects of extrinsic and intrinsic perturbations on the electronic structure of graphene: Retaining an effective primitive cell band structure by band unfolding. *Phys. Rev. B*, 89:041407(R), Jan 2014.

[16] R Maspero, S J Sweeney, and Marian Florescu. Unfolding the band structure of gaasbi. *Journal of Physics: Condensed Matter*, 29(7):075001, dec 2016.

[17] Yuji Ikeda, Abel Carreras, Atsuto Seko, Atsushi Togo, and Isao Tanaka. Mode decomposition based on crystallographic symmetry in the band-unfolding method. *Phys. Rev. B*, 95:024305, Jan 2017.

[18] S. Kurth and F. G. Eich. Overhauser's spin-density wave in exact-exchange spin-density functional theory. *Phys. Rev. B*, 80:125120, Sep 2009.

[19] Eli Rotenberg, B K Freelon, H Koh, A Bostwick, K Rossnagel, Andreas Schmid, and S D Kevan. Electron. *New J. Phys.*, 7:114–114, apr 2005.

[20] Eric Fawcett. Spin-density-wave antiferromagnetism in chromium. *Rev. Mod. Phys.*, 60:209–283, Jan 1988.

[21] R. Pynn, W. Press, S. M. Shapiro, and S. A. Werner. Second and third harmonics of the spin density wave in chromium metal. *Physical review*, 13(1):295–298, 1 1976.

[22] M Mori and Y Tsunoda. Searching for charge density waves in chromium. *Journal of Physics: Condensed Matter*, 5(7):L77, feb 1993.
